# Neural Mechanisms of the Transformation from Objective Value to Subjective Utility: Converting from Count to Worth

**DOI:** 10.3389/fnins.2016.00507

**Published:** 2016-11-09

**Authors:** Yoanna A. Kurnianingsih, O'Dhaniel A. Mullette-Gillman

**Affiliations:** ^1^Department of Psychology, National University of SingaporeSingapore, Singapore; ^2^Neuroscience and Behavioral Disorders Program, Duke-NUS Medical SchoolSingapore, Singapore; ^3^Singapore Institute for Neurotechnology (SINAPSE), National University of SingaporeSingapore, Singapore

**Keywords:** value, utility, gains, losses, risk, preferences, decision-making, executive function

## Abstract

When deciding, we aim to choose the “best” possible outcome. This is not just selection of the option that is the most numerous or physically largest, as options are translated from objective value (count) to subjective value (worth or utility). We localized the neural instantiation of the value-to-utility transformation to the dorsal anterior midcingulate cortex (daMCC), with independent replication. The daMCC encodes the context-specific information necessary to convert from count to worth. This encoding is not simply a representation of utility or preference, but the interaction of the two. Specifically, the relationship of brain activation to value is dependent on individual preference, with both positive and negative slopes across the population depending on whether each individual's preference results in enhancement or diminishment of the valuation. For a given value, across participants, enhanced daMCC activation corresponds to diminished subjective valuation, deactivation to enhanced subjective valuation, and non-modulated activation with non-modulated subjective valuation. Further, functional connectivity analyses identified brain regions (positive connectivity with the inferior frontal gyrus and negative connectivity with the nucleus accumbens) through which contextual information may be integrated into the daMCC and allow for outputs to modulate valuation signals. All analyses were replicated through an independent within-study replication, with initial testing in the gains domain and replication in the intermixed and mirrored losses trials. We also present and discuss an ancillary finding: we were unable to identify parametric value signals for losses through whole-brain analyses, and ROI analyses of the vmPFC presented non-modulation across loss value levels. These results identify the neural locus of the value-to-utility transformation, and provide a specific computational function for the daMCC in the production of subjective valuation through the integration of value, context, and preferences.

## Introduction

In decision making, we strive to choose the best option, but this is not simply the selection of the physically largest option or the option with the most numerous items. We determine the subjective valuation of each option (utility or worth) by integrating context and history. Such subjective valuation varies across individuals and also within individuals over time. For example, whereas a hungry person has a high subjective value for food, a satiated person may find the same food neutral or aversive. In monetary decision making, individuals often show widely varied subjective valuation, as demonstrated in individual responses to risky gambles (uncertain outcomes). Individuals may be willing to pay quite different prices for a lottery ticket with an expected value of $10 (50% chances of $20 or $0). While most individuals place their subjective valuation below the expected value (risk aversion), others enhance the subjective value (risk seeking). The degree and direction of their value modulation describes the specific value-to-utility transformation each participant is performing.

This is the first study to identify a brain region that encodes the information necessary to perform the value-to-utility transformation—the interaction of valuative signals and individual preferences. This experiment extends prior studies that identified brain regions with parametric encoding of value (for recent meta-analyses see Bartra et al., 2013; Clithero and Rangel, 2014), or modulation that tracks individual preference values (for examples, see Huettel et al., 2006; Kable and Glimcher, 2007).

The value-to-utility function is defined by two pieces of information: the value under consideration and the preference of the individual considering it. We opted to use money as our valuative/counting system for its cardinality, and risky decision making as our preference domain as it has been extensively studied behaviorally and shows significant variability across individuals (including both enhancement or diminishment, as in the prior example; see Kurnianingsih and Mullette-Gillman, 2015).

The value-to-utility transformation is a necessary process for determining the context-specific utility of many of the possible outcomes that we consider everyday. One clear example is within numeric systems, which veridically convey objective information between individuals and across neural systems. We all agree that zero pencils means there are none and that 10 pencils are twice as many as 5. Critically, the objective values of numeric systems (counts) must be converted to subjective values (utility) in order for us to be able to identify and select the option with the highest worth. As monetary systems are numeric, economic decision making that includes individual preferences (risk, temporal discounting, etc.) requires value-to-utility transformations in order to determine subjective valuation. However, the need for value-to-utility transformations is not limited to numeric systems, but extends across other systems in which consideration of possible outcomes requires mnemonic recollection of abstracted concepts that must incorporate context to determine their utility—a value-to-utility transformation. While it is theoretically possible that the brain could separately store each object-utility pair, the storage demands are biologically implausible and a more efficient process would be to encode these abstractions and perform value-to-utility transformations as necessary to determine the specific utility for the context under consideration. In this manner, the concept of a glass of water would be stored without a specific subjective valuation, which would be computed as needed for its contextually varied utilities (high utility in a desert, low utility on a sinking ship).

To identify the brain regions that contain the information necessary to perform value-to-utility transformations, we sought to identify a region that encodes the interaction between value and individual preference. To do so, we employed a precise computational model that examined nine levels of objective value across the idiosyncratic subjective valuation of each participant. This model leverages cardinal within-subject valuation and between-subject variability in risk preferences to construct the presented value-to-utility function of each participant and then identify a brain region that encodes these functions across participants.

As there is no consensus on the specific format in which the brain encodes value signals (what specific dimensions are encoded), a potential danger is that we could select to use a theoretical formulation that was not actually present in the brain. To ensure the viability of the value formulation utilized, our first step was to examine how well a few candidate formulations were represented within the ventromedial prefrontal cortex (vmPFC), a region that is reliably found to encode valuative signals (for recent meta-analyses see Bartra et al., 2013; Clithero and Rangel, 2014). We then selected one of these formulations for our principle analyses. Subsequently, we performed *post-hoc* analyses demonstrating that the results generalize across the value formulations found to be represented within the vmPFC.

This study extends prior experiments that examined the neural encoding of valuation, to identify the brain regions that encode the information necessary to transform value signals from objective to subjective representations (value to worth). The critical difference between models to identify valuative regions and models to identify regions that encode the information necessary for the value-to-utility transformation are the neural responses of the brain region across subjects. Prior fMRI studies seeking to identify valuative regions have used analytic models that identify brain regions whose activation is correlated with the value presented on each trial, with a slope that is the *same across participants*. Our value-to-utility transformation analyses identify brain regions whose activation is correlated with the value on each trial, but with a slope that *varies across participants as a function of each individual's idiosyncratic risk preference.* This difference greatly enhances the specificity of the computational model, resulting in mathematical orthogonality to prior studies that have separately investigated the neural encoding of value or risk preferences alone. Therefore, the results of prior studies that have identified brain regions that encode value or risk preference alone are not predictive of the brain regions in which we may expect to identify the interaction of the two (although they may be likely to interact in some manner).

Previous fMRI studies have indicated numerous brain regions involved in value processing, including the nucleus accumbens, striatum, and vmPFC (for recent meta-analyses see Bartra et al., 2013; Clithero and Rangel, 2014). Several brain regions have been identified to show variation in decision-related activation correlated with individual differences in risk preferences, notably including the parietal cortex and lateral prefrontal regions (Huettel et al., 2006; Christopoulos et al., 2009). Our predictions were that the value-to-utility information would be encoded in a brain region that can act as an integrative hub across these brain regions, integrating executive processes in lateral prefrontal regions with ventral valuative regions, with notable candidates in the caudate and cingulate cortex.

Our results demonstrate that the dorsal anterior midcingulate cortex (daMCC) encodes the information necessary to perform the value-to-utility transformation—the interaction of valuative and preference information. Critically, the distribution of slopes can account for both presented enhancement and diminishment of subjective value (utility/worth) across participants. In addition, functional connectivity analyses identified brain regions that may be involved in the value-to-utility transformation (positive connectivity with the inferior frontal gyrus and negative connectivity with the nucleus accumbens), such as providing contextual information for integration in the daMCC and outputs that could modulate known valuative signals.

Of note, our methodology features independent within-study replication. Neuroimaging analyses were first performed on gains trials and then replicated within losses trials, allowing for almost perfectly matched and intermixed tasks—differing only by sign and individual preferences (uncorrelated across domains). *Post-hoc* analyses also examined the generalization of our results across variations in our model. These features provide a high level of confidence in our results—the daMCC encodes the interaction between valuative information and individual preferences, the information necessary to perform the value-to-utility transformation.

## Methods

### Participants

Thirty healthy subjects (15 males, *mean* ± *SD* age = 22 ± 1.74 years old) were recruited from the National University of Singapore as participants in this study. They were all right-handed with no history of neurological or psychiatric disorders. Participants provided written informed consent under a protocol approved by the National University of Singapore Institutional Review Board. fMRI scanning was conducted in the Duke-NUS Medical School, Singapore.

### Study procedure

Participants participated in two sessions: a behavioral session followed by an fMRI session (8–152 days in between). During the behavioral session participants performed the risky monetary decision task using a computer outside the scanner (results published in Kurnianingsih and Mullette-Gillman, 2015). Participants whose choices did not rely on confounding behavioral choice patterns (such as always choosing the risky or certain option) were invited to return for the fMRI session. During the fMRI session, participants performed the risky monetary decision task inside the MRI scanner.

### Risky monetary decision task

**Figure 1:**
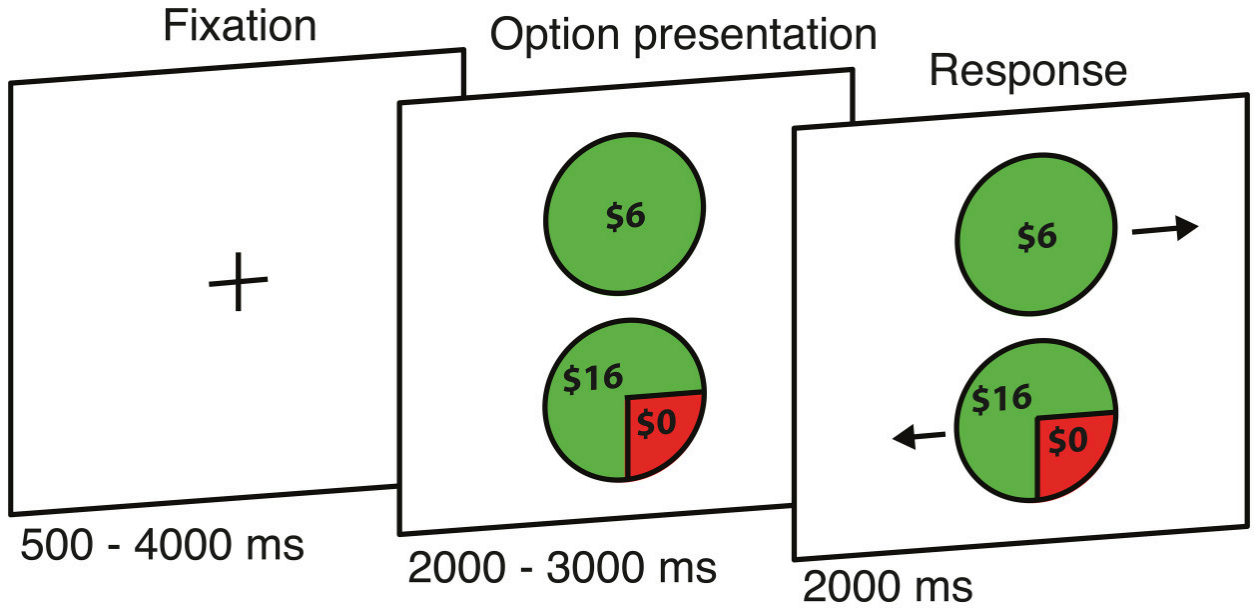
**Risky monetary decision task**. Each trial begins with the presentation of two options, followed by an arrow appearing at the side of each option (position randomly interchanged) to indicate which button should be pressed to select that option.

We used a modified version of a risky monetary decision task (Kurnianingsih and Mullette-Gillman, 2015), with an equal number of trials evaluating the gains and losses domains, randomly intermixed. On each trial, participants chose between a gamble and a certain option (270 trials) or between two certain options (74 trials) (Figure 1). For gains, the trial matrix was made up of five different values of the certain option (V_Certain_){$3, $4, $5, $6, $7}, with the gamble option constructed from three probabilities of winning (pWIN){25, 50, 75%} and nine relative expected values between the certain and gamble options (rEV = EV_Gamble_/ V_Certain_){0.25, 0.50, 0.66, 0.80, 1.0, 1.25, 1.50, 2.0, 4.0}. For certain vs. certain trials in the gains domain, the trial matrix was constructed from five different certain values for the first option (V_Certain1_){$3, $4, $5, $6, $7}, and the values of the second option were calculated based on the combination of V_Certain1_ and rEV (V_Certain2_ = rEV × V_Certain1_), with nine different relative expected values (rEV){0.25, 0.50,.66, 0.80, 1.0, 1.25, 1.50, 2.0, 4.0}. Note, that this process results in a small number of duplicate trials, which were not included. The losses trials mirrored the gains trials, with the sign of the values adjusted to negative. Behavioral data collection and analyses were achieved using Matlab R2010B (Mathworks, Natick, MA) with Psychtoolbox (www.psychtoolbox.org) (Brainard, 1997) for trial presentation. No trials were resolved before the end of the experimental session, to prevent feedback from altering subsequent behavior (learning). At the beginning of the session participants were informed that their payment would be determined by the resolution of one gains trial and one losses trial randomly selected from each run at the end of the session (a total of 10 trials, from four actual runs and one practice run).

#### fMRI task design

Each participant underwent four runs; each run consisted of 43 gains trials and 43 losses trials, lasting for 9 min and 22 s. The trials were divided equally into four runs, with trial order randomized independently for each participant. Each trial began with the presentation of the two options (2000–3000 ms) followed by arrows appearing at the left or right side of each option (2000 ms). Participants had 2000 ms to respond, by pressing the key on the button box that corresponded to the direction of the arrow presented beside their preferred option (position randomly interchanged). After a response was made, the screen presentation was immediately replaced by a fixation cross for the remainder of the 2000 ms and then continued during the inter-trial intervals (500–5500 ms). In other words, quick responses do not reduce the duration of the run but increase the baseline time (improving the fMRI signal to noise ratio). All trials and time intervals within each block were fully randomized for each participant.

To ensure that participants were incentivized to not miss trials, they were informed that if a missed trial was selected for resolution toward their payment, an option would be randomly selected and an additional penalty of −$2 applied. Any such missed trials were excluded from analyses. On average, participants missed .18 gains trials (*SD* = 0.52, with a range of 0–3, *mean* ± *SD* = 0.43 ± 1.21%) and .41 losses trials (*SD* = 0.87, with a range of 0–5, *mean* ± *SD* = 0.96% ± 2.02%). Missed trials were excluded from analyses.

We excluded one run of one participant from fMRI analyses due to a technical problem during data acquisition.

#### Practice task

Before entering the MRI scanner, participants were given a set of computerized practice trials. The practice task consisted of 60 risky vs. certain trials, constructed from two possible rEV{0.33, 3.0} and three possible probabilities of winning (pWIN){25, 50, 75%} in both the gains and losses domains. Trials were presented as they would be inside the MRI scanner, but were not included in behavioral analyses.

#### Behavioral analysis. quantifying risk preferences

We quantified risk preferences separately for the gains and losses domains, by using these power functions (Tymula et al., 2013; Kurnianingsih and Mullette-Gillman, 2015):

$$\begin{array}{l} \text{ For gains }\left(\text{if V}>0\right):\text{ SV}=\text{pWIN}\times {\text{V}}^{\alpha .}\\ \text{For losses }\left(\text{if V}<0\right):\text{ SV}=-\left(1-\text{pWIN}\right)\times {\left(-\text{V}\right)}^{\alpha .} \end{array}$$

where SV is the subjective value (utility) of the gamble, pWIN is the probability of receiving the better outcome of the option (assuming linear probability weighting), V is the objective value of the option (which is the nominal value that was presented), and α is the degree of the power function curvature that represents the degree each participant modulates the values of the options. In the gains domain, an α < 1 indicates value diminishment (SV < V, risk averse), an α = 1 indicates the absence of value modulation (SV = V, risk neutral), and an α > 1 indicates value enhancement (SV > V, risk seeking). Due to the negative signs in the losses domain, the opposite applies. In the losses domain, an α < 1 indicates value enhancement (risk seeking), an α = 1 still indicates the absence of value modulation (risk neutral), and an α > 1 indicates value diminishment (risk averse).

In order to determine participant's risk preference, participant's choice data were fitted using maximum likelihood with a probability choice function:

$$\begin{array}{l} \text{Probability of choosing the gamble option}=\frac{1}{1+e^{-\left(SV_{G}-SV_{C}\right)}} \end{array}$$

Where SV_C_ is the subjective value of the certain option and SV_G_ is the subjective value of the gamble option.

#### MRI data acquisition

MR images were acquired on a 3T Siemens Tim Trio (Siemens, Erlangen, Germany). Visual stimuli were back-projected onto a screen positioned behind the scanner bore (Epsom EMP1715, 800 × 600 pixels, 60 Hz). Four runs of 283 volumes each were acquired using a gradient echo-planar imaging (EPI) sequence with the following parameters: repetition time (TR) = 2000 ms; echo time = 30 ms; flip angle = 90 degrees; field-of-view (FoV) = 192 × 192 mm; matrix size = 64 × 64 with resolution of 3 × 3 mm. Each volume consisted of 36 slices collected in an interleaved ascending manner. The slices were aligned to the anterior commissure-posterior commisure (AC-PC) plane. We also obtained a T1-weighted coplanar image and a high-resolution T1-weighted anatomical volume (1 × 1 mm) acquired using a 3D-MPRAGE sequence to assist with image co-registration.

#### Image preprocessing and statistical analysis

Image processing and statistical analysis were conducted using FSL Version 5.0.2.2 FEAT Version 6.0 (Brainard, 1997) and MATLAB R2010B (Mathworks, Natick, MA), with visualization of neural results using MRIcron (Rorden et al., 2007) and MRIcroGL (http://www.cabiatl.com/mricrogl/). A total of 10 volumes were discarded to ensure sufficient time for the scanner signal to reach equilibrium. Brain extraction of the functional and anatomical images was performed with FSL's Brain Extraction Tool (BET) (Smith, 2002). Functional runs were spatially smoothed using a 5 mm full-width-half-maximum Gaussian kernel, filtered in the temporal domain using a high pass filter cutoff of 30 s and motion corrected using MCFLIRT (Jenkinson et al., 2002). Translation movements were <1 voxel for all runs of all subjects. Functional images were normalized using FLIRT (Jenkinson and Smith, 2001; Jenkinson et al., 2012) by estimating the transform from individuals' T1-weighted coplanar (6 degree-of-freedoms) and high-resolution T1-weighted anatomical image (7 degree-of-freedoms); the resulting data were then aligned into MNI standard space (12 degree-of-freedoms). All reported neuroimaging main effects and contrasts unless specified utilize a height threshold of *z* > 2.3 and a standard cluster probability of *p* < 0.05. We note, as recently significant concerns have arisen over the assumptions of statistical cluster-correction software (Eklund et al., 2016), that this same paper indicates that the software package and analyses we used, FSL with FLAME 1 (a Bayesian mixed effects approach with randomized events), do not result in elevated false positive rates. In addition, our experimental design, featuring a highly specific analyses and within-study replication, safeguard us against growing concerns of the rate of false-positive findings in the field of cognitive neuroscience (Szucs and Ioannidis, 2016).

#### General linear model (GLM). GLM1

The base GLM model had five predictors, each convolved using a double gamma hemodynamic response function. This model is a basic 2 × 2 design (risky/certain × gains/losses), with box-car encoding of the decision phase (option onset to button press) for each of four types of trials (#1 gains trials with risky vs. certain options regressor, #2 losses trials with risky vs. certain options regressor, #3 gains trials with certain vs. certain options regressor, and #4 losses trials with certain vs. certain options regressor), and a nuisance regressor for button presses (regressor #5, 500 ms starting at press).

#### GLM2 a, b, and c

These three additional models investigated value signal coding across trials in varied theoretical formulations of [Value]. The [Value] formulations examined were rEV (relative expected value; expected value of the gamble divided by the value of the certain option), CV (chosen value; the expected value of the chosen option), and rCV (relative chosen value; the expected value of the chosen option divided by the expected value of the unchosen option). Separate GLMs were performed to examine each of [Value] formulations (*GLM2 a, b* and *c*, respectively), as the formulations are correlated across trials (Table S1).

Each of the *GLM2* (*a, b*, and *c*) models featured the addition of six additional predictors, each convolved with a double gamma hemodynamic response function: #6 parametric regressor of [Value] in gains trials with risky vs. certain options, #7 parametric regressor of [Value] in losses trials with risky vs. certain options, #8 parametric regressor of pWIN in gains trials with risky vs. certain options, #9 parametric regressor of pWIN in losses trials with risky vs. certain options, #10 parametric regressor of [Value] in gains trials with certain vs. certain options, and #11 parametric regressor of [Value] in losses trials with certain vs. certain options. Each of these regressors (#6 to #11) encoded the entire decision phase, from onset of option presentation to the button press response. The pWIN regressors were both orthogonalized with respect to the risky vs. certain options [Value] regressor within their respective domains.

To identify the neural encoding of the value-to-utility transformation, covariate analyses were performed separately for the gains and losses domains by including each individual's risk preference values for each domain as a between-subject covariate into the GLM model. Beta values were extracted from a daMCC ROI (**Figures 3C**, **4C**), constructed through a conjunction analysis of the separate gains and losses covariate analyses.

#### GLM3

This categorical model allows for the extraction of the actual functional neural encoding of the rEV value signal. This model consisted of *GLM1* plus 18 additional categorical regressors, encoding the nine levels of rEV presented across trials (for each domain). The risky vs. certain trials were grouped according to their rEV (2 domains × 9 rEVs) and each rEV value was represented by a boxcar task regressor encoding the entire decision phase, from option presentation to response button press, and convolved with a double gamma hemodynamic response function.

#### Psychophysiological interaction (PPI) analysis

This analysis was performed to examine brain regions that have task-related functional connectivity with the daMCC during the decision period. We utilized the daMCC ROI produced as the conjunction of the voxels found to contain the value-to-utility transformation information across both the gains and losses domains. For each individual, an average time series of the voxels within the daMCC seed ROI was computed from the voxels for each trial type. A GLM model was estimated by adding in eight additional regressors to *GLM1* model (two additional regressors for each trial type). These additional regressors were the time course of the seed ROI averaged across the ROI voxels, and the interaction between the time course regressor and boxcar trial type regressor for each trial type.

## Results

### Behavior

Risk preferences were quantified for each participant, separately for the gains and losses domains. In the gains domain, on average participants were risk averse (utility less than value, *mean* ± *SD* α = 0.84 ± 0.21). In the losses domain, on average participants were risk neutral (*mean* ± *SD* α = 1.04 ± 0.26). There was no significant correlation between individual risk preferences across the gains and losses domains (*r*_28_ = 0.26, *p* = 0.16), concurring with recent studies (Kurnianingsih and Mullette-Gillman, 2015; Kurnianingsih et al., 2015; Mullette-Gillman et al., 2015a,b). For further consideration of this and additional behavioral analyses we point the reader to Kurnianingsih and Mullette-Gillman (2015), which features a larger sample (including the individuals in this study) performing the same task with slightly more trials (30 additional trials by sampling two additional levels of rEV).

### Contrasting decision types within and across gains and losses

We determined gross differences in brain activations between the trials in which participants chose between risky and certain options and those trials in which they chose between two certain options, examined separately within the gains and losses domains (Table S2). We also identified differences in the brain activations between gains and losses for each trial type (Table S3).

### Identifying the neural encoding of value signal for gains

**Figure 2:**
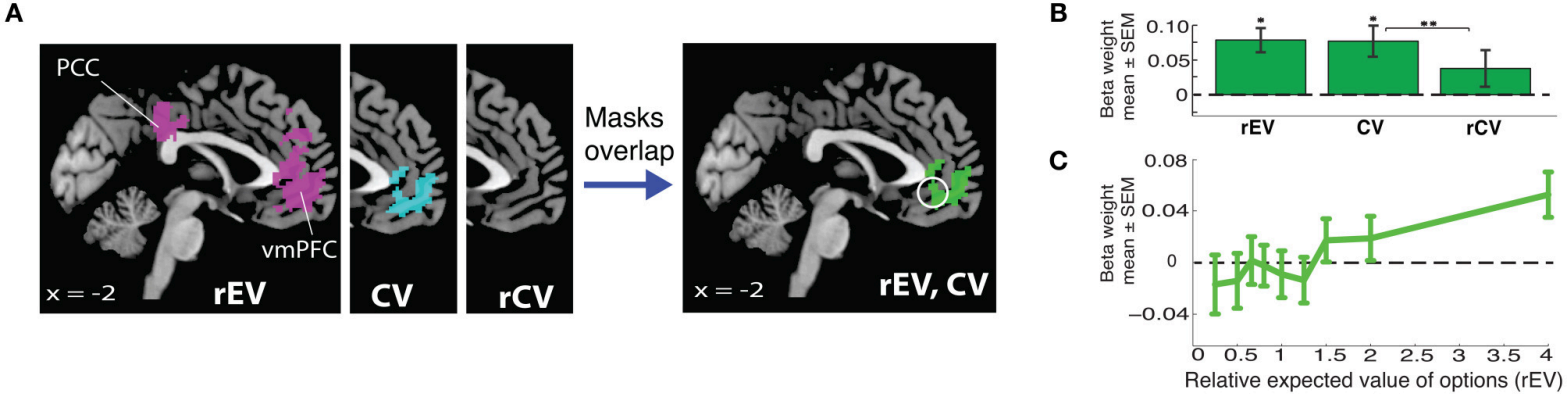
**Neural encoding of value signals for gains**. **(A)** Whole brain analyses localizing value signals with three different value regressors (relative expected value, rEV; chosen value, CV; relative chosen value, rCV). The white circle indicates the unbiased 10 mm spherical vmPFC ROI (*x* = −2, *y* = 40, *z* = −8). **(B)** vmPFC ROI analyses demonstrate the strength of the encoding for each of the three tested formulations of trial value (^*^significantly different from 0; ^**^significantly different from each other). **(C)** Extracted functional relationship between vmPFC activation and each gain rEV category, extracted from the vmPFC ROI.

Numerous studies have shown that activation within the vmPFC is parametrically modulated by the value presented on each trial (Bartra et al., 2013; Clithero and Rangel, 2014). While this result is robust across numerous studies, it is still unclear how value is specifically represented in the brain. As we sought to utilize a value signal as a component of our localization of the value-to-utilty information, we first tested three different formulations to parameterize value on each trial, in order to identify the one most robustly represented in the vmPFC during decision making. These three formulations were: 1) the ratio of the expected value of the gamble to the value of the certain option (EV_gamble_/V_certain_, which we will refer to as rEV); 2) the expected value of the chosen option (which we will refer to as CV); and 3) the ratio of the expected value of the chosen option to the expected value of the unchosen option (EV_chosen_/EV_unchosen_, which we will refer to as rCV). Each of these value formulations was tested with an independent general linear model (GLM), examining the whole-brain encoding of parametric value signals within the trials in which participants chose between risky and certain options in the gains domain (Figure 2; Table S4; utilizing *GLM2*).

We found clear evidence that the vmPFC encodes parametric value signals for both the rEV and CV value formulations, with large overlap in the voxels identified for each of the two models (Figure 2A). We compared the quality of the fits for these formulations by extracting the beta values for each parametric value regressor from an unbiased 10 mm spherical vmPFC region-of-interest (ROI) (*x* = −2, *y* = 40, *z* = −8), centered on the peak coordinate of parametric value signals reported in a meta-analysis study by Bartra et al. (2013) (Figure 2B). The strengths of the representations of the rEV and CV value formulations were not significantly different from one another (*t*_29_ = 0.15, *p* = 0.88). The rCV formulation resulted in no significant voxels at the whole-brain level, and the ROI analyses confirmed that the strength of the encoding was not significantly greater than zero (*t*_29_ = 1.46, *p* = 0.15) and was significantly less than the rEV and CV formulations (rEV *t*_29_ = 2.03, *p* = 0.051; CV *t*_29_ = 2.90, *p* = 0.007).

Given the design of our task, with 15 trials for each of the 9 levels of the rEV formulation, we were able to visualize the actual neural encoding of the rEV formulation within the vmPFC ROI by extracting the beta values for each level of the rEV formulation (utilizing *GLM3*). The encoding of gains value signals in the vmPFC demonstrated a clear positive relationship between value and brain activation (Figure 2C). As the rEV and CV formulations equally well captured the value signals within the vmPFC, we chose to first focus analyses on the rEV factor. *Post-hoc* analyses demonstrate the congruence of results across both the rEV and CV formulations (below).

### Identifying the neural encoding of the value-to-utility transformation, in gains

**Figure 3:**
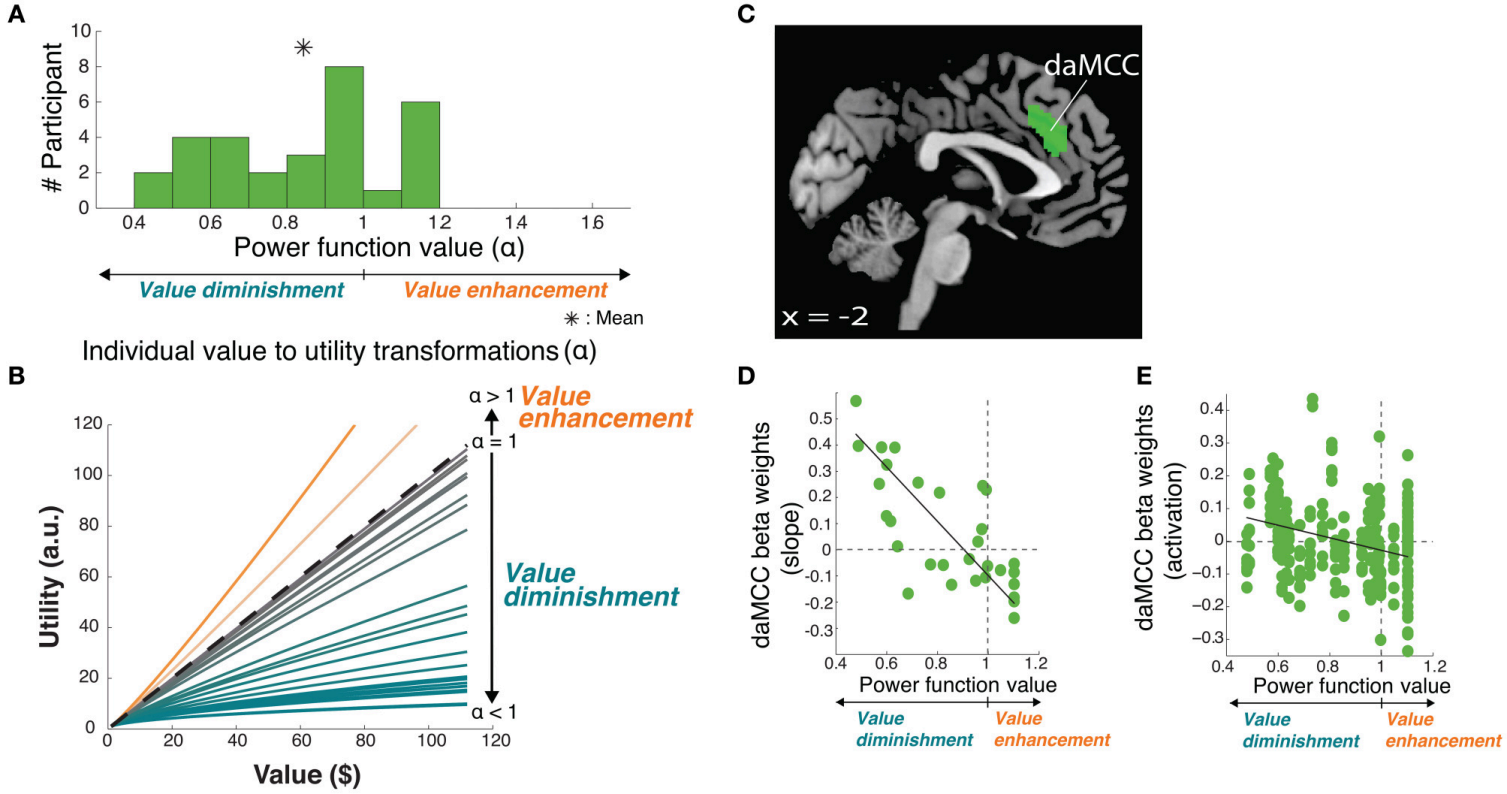
**Value-to-utility transformation for gains**. **(A)** Distribution of risk preferences in the gains domain as measured by power function values (α). **(B)** Distribution of individual value-to-utility transformations revealed by individual preferences. **(C)** Neural regions encoding the value-to-utility transformation. **(D)** Relationship between individual preferences and extracted daMCC beta values (slopes) from the covariate analysis. Note that the points are extracted from a whole-brain identified ROI, and are meant to illustrate the already-identified relationship. **(E)** Relationship between individual preferences and extracted daMCC betas values (activation) from the categorical model.

The principle purpose of this project was to localize the neural mechanisms of the value-to-utility transformation. To do so required precisely quantifying the value-to-utility transformation that each participant was performing, as captured based on their risk preference as expressed by their choices [modeled as the degree of curvature (α) of their individual utility function, a power function (Tymula et al., 2013)]. In the gains domain, participants were on average risk averse (utility less than value, *mean* ± *SD* α = 0.84 ± 0.21), with a wide range of preference values including individuals who were risk seeking (utility greater than value, α > 1) (Figure 3A).

Armed with the behaviorally derived quanitification of each individual's value-to-utility transformation, we sought the neural instantiation by covarying the value on each trial (here, the rEV regressor; below, *post-hoc* analysis of the CV regressor) against each individual's risk preference. This between-subject covariate analysis identifies neural regions that encode a linear value signal across trials, whose slope varies across participants (positive to negative) based on the degree and direction of the value-to-utility transformation each individual is using to make their choices (Figure 3B). Whole-brain analyses revealed a significant fit to this function in voxels within the dorsal anterior midcingulate cortex (daMCC) (Bush, 2009) (Figure 3C; Table 1), in a region also referred to as the anterior midcingulate cortex (aMCC) (Vogt, 2005) or more generally as part of the dorsal medial prefrontal cortex (dmPFC).

**Table 1:** **Neural encoding of the value-to-utility transformation across gains and losses**.

	**# Voxels**	**Regions**	**Hemisphere**	**Peak Coordinates**			***z-stat***
				***x***	***y***	***z***	
Gains rEV × α	794	Paracingulate Gyrus (referred to as daMCC, aMCC, or dmPFC)	L	−2	32	26	3.91
			R	2	26	36	3.87
			mid	0	32	30	3.83
	301	Supramarginal Gyrus	R	46	−44	56	3.21
			R	44	−42	46	3.1
			R	52	−46	54	2.99
Losses rEV × α	1055	Frontal Orbital Cortex	L	−44	30	12	3.32
		Frontal Pole	L	−28	54	0	3.83
		Inferior Frontal Gyrus	L	−42	14	24	3.29
	683	Paracingulate Gyrus (referred to as daMCC, aMCC, or dmPFC)	L	−6	28	26	3.73
			L	−2	22	38	3.57
		Superior Frontal Gyrus	L	−14	16	48	3.64
	482	Insular Cortex	L	−34	18	−6	3.79
		Frontal Orbital Cortex	L	−30	30	−10	3.56
			L	−44	28	−20	3.15
	362	Postcentral Gyrus	L	−42	−22	30	3.23
			L	−50	−14	38	3.2
		Precentral Gryus	L	−48	−14	46	3.08
	1645	Lingual Gyrus	R	20	−56	2	4.06
		Occipital Fusiform Gyrus	R	34	−66	−22	3.85
		Hippocampus	R	22	−26	−10	3.78
	1545	Supramarginal Gyrus	L	−28	−64	4	3.94
			L	−4	−62	−8	3.68
			L	−24	−56	−24	3.6
	675	Lateral Occipital Cortex	R	18	−68	58	3.77
			R	8	−72	60	3.18
		Procuneous Cortex	R	14	−64	33	3.19
	763	Lateral Occipital Cortex	L	−28	−80	42	3.33
			L	−14	−74	58	3.19
			L	−10	−70	60	3.12

rEV, relative expected value; α, alpha; daMCC, dorsal anterior midcingulate cortex; aMCC, anterior midcingulate cortex; dmPFC, dorsomedial prefrontal cortex.

Regions are labeled based upon their Harvard-Oxford Atlas designations, with parenthetical inclusion of labels from text.

The coordinates for the three peak activations are provided for each cluster, in MNI space (in mm).

To examine the nature of the relationship between these indicated voxels and the value-to-utility transformation, we performed ROI analyses on the daMCC cluster to illustrate the whole-brain identified linear relationship between individual subjective value modulation and the extracted beta values from the covariate analysis (*r*_28_ = −0.73, *p* < 0.0001) (Figure 3D). As the extracted beta values are derived from a parametric regressor (9 levels of rEV) they indicate the slope of the relationship between that regressor and daMCC activation. Interestingly, the resulting distribution of beta values is zero-centered with positive betas (slopes) for value diminishment and negative betas (slopes) for value enhancement.

Given the complexity of the covariate analyses, we sought to clearly describe the relationship between daMCC activation and the value-to-utility transformation—how modulated daMCC activation/deactivation corresponds to reduced/enhanced subjective valuation. We anticipated finding the same zero-centered negative relationship as apparent in the covariate ROI analysis. To confirm this relationship, for each participant we extracted their daMCC activation level for each of the 9 levels of the categorical rEV regressors (from *GLM3*), and across participants, regressed these values against each individual's risk preference (a 270 point fit). The solution was significant [*F*_(1, 268)_ = 13.02, *p* < 0.001] with a slope of −.13 (*SEM* = 0.036) and an intercept of.11 (*SEM* = 0.031). The negative slope confirms the overall relationship between daMCC activation and the value-to-utility transformation—the more activated the daMCC is the more diminished the subjective value will be (and the reverse). Further, based on the intercept we can calculate the daMCC activation at risk neutrality (α = 1) to be −0.02, which is within 1 *SEM* from zero, confirming the zero-centeredness of this function (Figure 3E).

Enhanced activation in the daMCC corresponds to diminished subjective valuation, deactivation of the daMCC corresponds to enhanced subjective valuation, and baseline daMCC corresponds to non-modulation of subjective valuation (utility = value). This near-zero-centered bi-directional function provides a perfect substrate for the value-to-utility transformation.

### Replicating the neural encoding of the value-to-utility transformation, in losses

**Figure 4:**
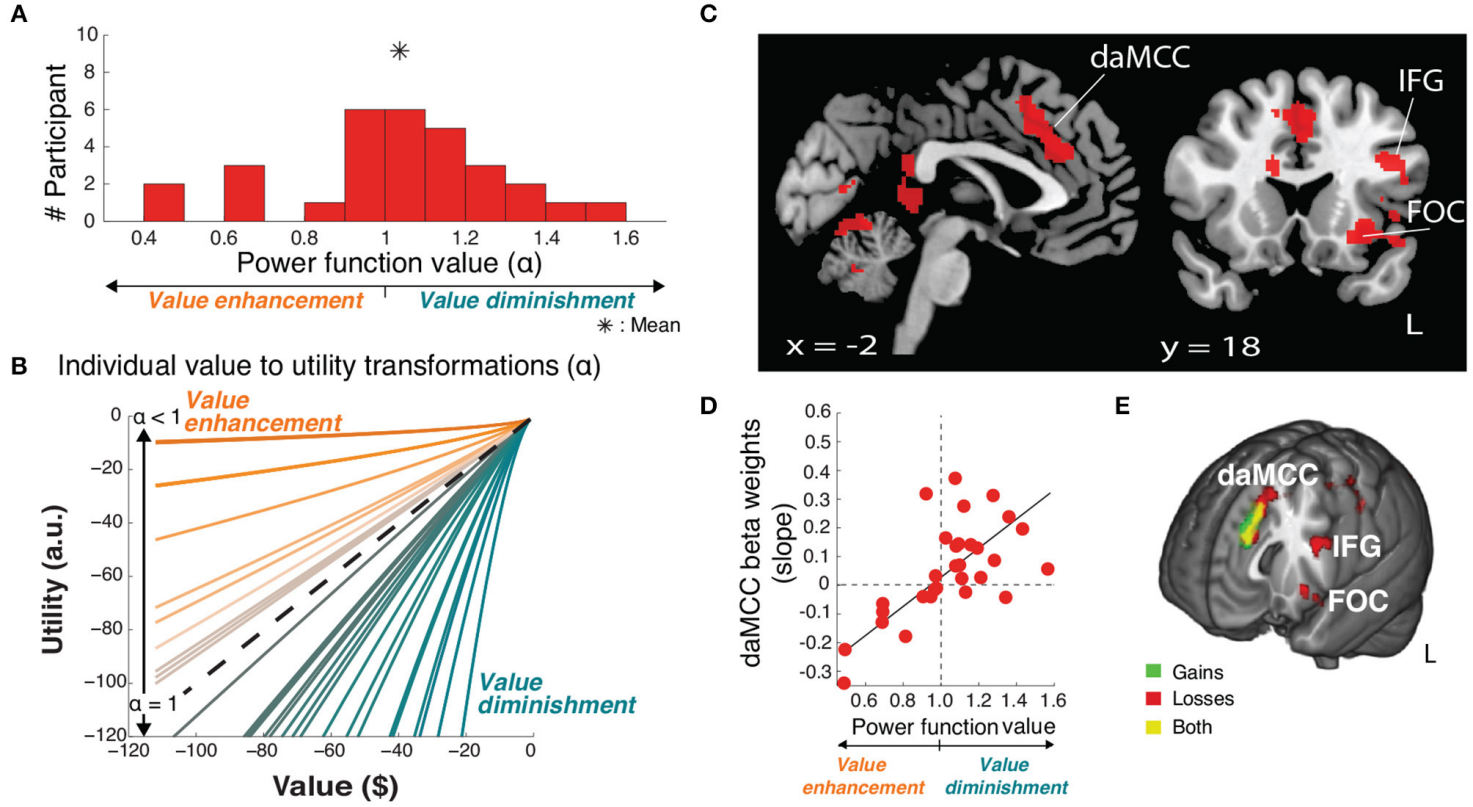
**Value-to-utility transformation for losses**. **(A)** Distribution of risk preferences in the losses domain as measured by power function values (α). **(B)** Distribution of individual value-to-utility transformations revealed by individual preferences. **(C)** Neural regions encoding the value-to-utility transformation. **(D)** Relationship between individual preferences and extracted daMCC beta values (slopes) from the covariate analysis. Note the inverted relationship between risk preference and value modulation across gains and losses (compare x-axes of Figures 3D, 4D) resulting in matched relations between daMCC beta values and the sign and degree of the value-to-utility transformation. **(E)** Overlap of regions encoding the value-to-utility transformation information across gains (Figure 3C) and losses (Figure 4C).

For the within-study replication, we repeated our analyses in the intermixed losses trials. On average, participants were risk neutral (*mean* ± *SD* α = 1.04 ± 0.26) (Figure 4A), with a range of preferences. There was no significant correlation between individual risk preferences across the gains and losses domains (*r*_28_ = 0.26, *p* = 0.16), concurring with recent studies (Kurnianingsih and Mullette-Gillman, 2015; Kurnianingsih et al., 2015; Mullette-Gillman et al., 2015a,b). We then replicated our analyses to identify the regions encoding the value-to-utility transformation, covarying the rEV value regressor by individual preferences (Figure 4B). These whole brain analyses identified a significant cluster within the daMCC encoding the value-to-utility transformation each individual was performing, replicating the results in the gains domain (Figure 4C, Table 1). ROI analysis within the daMCC revealed a clear linear relationship between value modulation (risk preference) and daMCC betas (losses: *r*_28_ = 0.64, *p* < 0.001) (Figure 4D). In near-perfect agreement with the function found for gains, positive daMCC betas correspond to value diminishment, negative betas correspond to value enhancement, and daMCC betas of zero correspond to no value modulation (α = 1).

Critically, these results confirm that the information for the value-to-utility transformation localizes to the daMCC (Figure 4E). Across both our initial investigation within the gains domain and the replication in the losses domain, we not only identified the same brain region across whole-brain analyses but also independently identified the same zero-centered functional relationship between the daMCC (slopes and activation) and the degree/direction of the value-to-utility transformation.

### Identifying brain regions involved in the value-to-utility transformation through functional connectivity analyses

**Figure 5:**
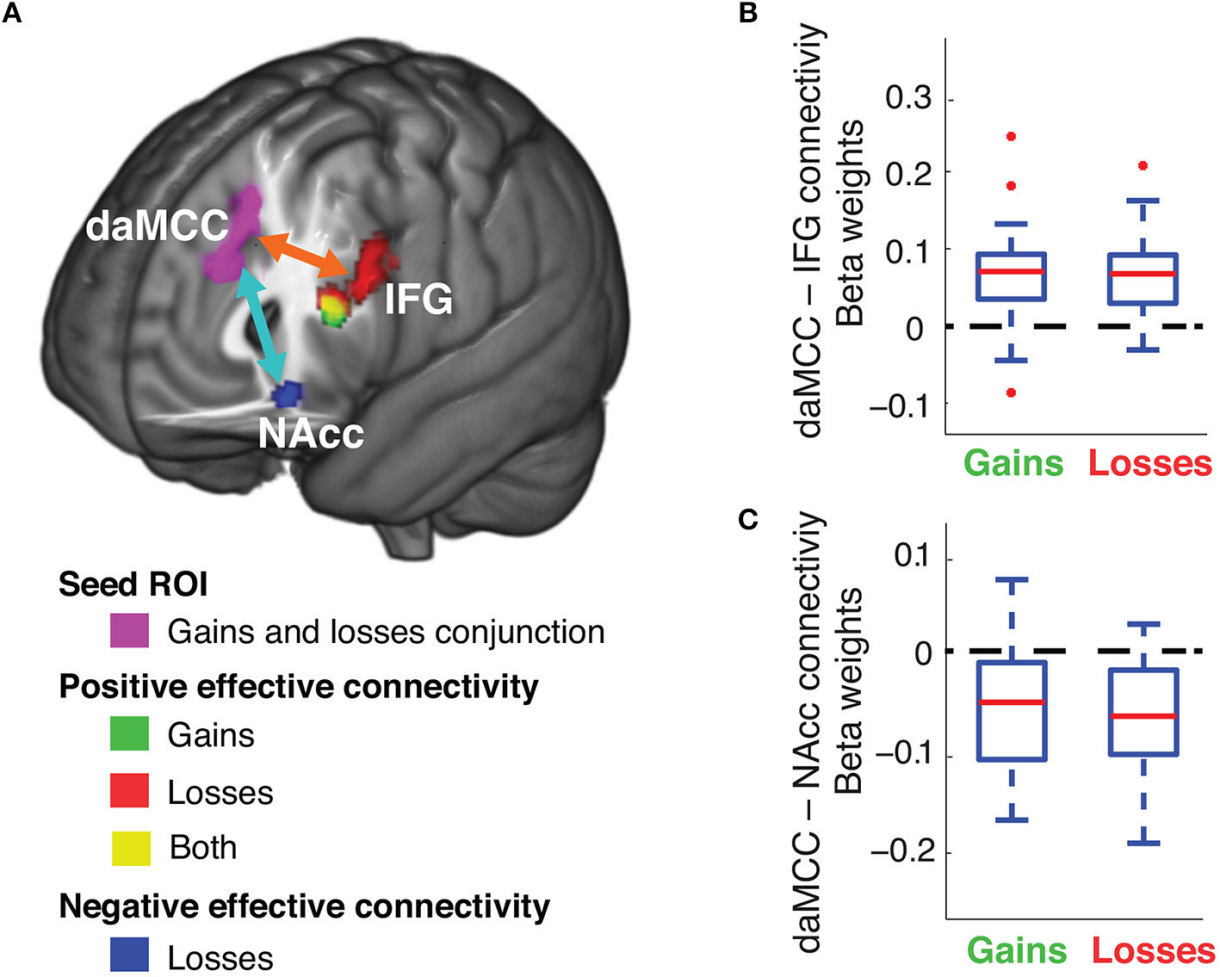
**Functional connectivity of the daMCC during decision making**. **(A)** Brain areas with decision-related functional connectivity to the daMCC. **(B)** Connectivity between daMCC and IFG, extracted from the conjunction of the significant voxels in the IFG for both gains and losses. **(C)** Connectivity between daMCC and NAcc, extracted from the losses NAcc region (for boxplot: red line is median, blue solid box indicates the 25–75 percentile, error bar indicates the range of non-outlier extreme values, “+” indicates outliers).

We examined the network of brain regions that communicate during the value-to-utility transformation, through functional connectivity analyses. To identify brain regions that have task-related functional connectivity with the daMCC during risky choices, psychophysiological interaction (PPI) analyses were performed separately for the decision periods of risky gains and losses trials, with a seed ROI from the daMCC (produced through a conjunction analysis across gains and losses, between Figures 3C, 4C). This analysis identified only two brain regions with significant functional connectivity with these daMCC voxels during the decision phase. The daMCC is positively connected to the left inferior frontal gyrus (IFG) and negatively connected to the nucleus accumbens (NAcc). Specifically, we found whole-brain significant positive connectivity to the left IFG for both gains and losses and negative connectivity to the NAcc for losses (Figure 5A and Table S5). We then performed ROI analyses on the identified IFG and NAcc voxels (IFG ROI derived as the conjunction of gains and losses, NAcc ROI as the voxels significant in losses). ROI analyses confirmed the whole-brain significances (Figures 5B,C) (gains IFG, *mean* ± *SD* beta:.067 ± 0.063, different from 0 *t*_29_ = 5.80, *p* < 0.0001; losses IFG, *mean* ± *SD*:.067 ± 0.055, different from 0 *t*_29_ = 6.67, *p* < 0.0001; losses NAcc, *mean* ± *SD*: −0.070 ± 0.056, different from 0 *t*_29_ = 6.78, *p* < 0.0001), and revealed significant functional connectivity between the daMCC and NAcc for gains (gains NAcc, *mean* ± *SD*: −0.056 ± 0.061, different from 0 *t*_29_ = 4.98, *p* < 0.0001).

### *Post-hoc* replication of the value-to-utility covariate analyses with alternative value and preference metrics

Our analyses demonstrate that the daMCC encodes the information necessary for the value-to-utility transformation. These results were obtained using a specific formulation for the value on each trial (rEV) and a common measure of individual risk preferences (α). To test the robustness and generalizability of the encoding of value subjectifcation in the daMCC, we repeated our analyses using an alternative formulation of value (chosen value, CV) and an alternative metric for quantifying individual risk preferences (risk premium, a linear formulation; Stanton et al., 2011; Kurnianingsih and Mullette-Gillman, 2015; Kurnianingsih et al., 2015; Mullette-Gillman et al., 2015a,b). These alternatives extend our analyses from simply across gains and losses to an additional 2 × 2 space across risk preferences (α and premium) and value formulations (rEV and CV). The goal is to examine whether the relationship between daMCC activation and value subjectification is robust across these alternative ways of quantifying components of value subjectification.

#### Alternative risk preference measure: risk premium

We additionally quantified each individual's risk premium using psychophysical indifference point analyses (for details see Kurnianingsih and Mullette-Gillman, 2015; Kurnianingsih et al., 2015; Mullette-Gillman et al., 2015a,b), which measures the degree and direction of value subjectification in a zero-centered multiplicative/linear form (as compared to the one-centered power function form of the α risk preference metric). Although these metrics have different theoretical assumptions, we find strong correlations between them in this study (gains: *r*_26_ = −0.74, *p* < 0.0001; losses: *r*_27_ = −0.45, *p* < 0.0001) and previous studies (Stanton et al., 2011; Kurnianingsih and Mullette-Gillman, 2015; Kurnianingsih et al., 2015; Mullette-Gillman et al., 2015a,b).

#### Alternative value regressor: chosen value

The CV regressor was constructed as the value of the chosen option on each trial, and was previously included in *GLM2b* and ROI analyses comparing the degree to which the vmPFC encodes different formulations of value regressors. Of note, these analyses indicated that the vmPFC equally encodes both the rEV and CV formulations of valuation.

#### ROI analyses

**Figure 6:**
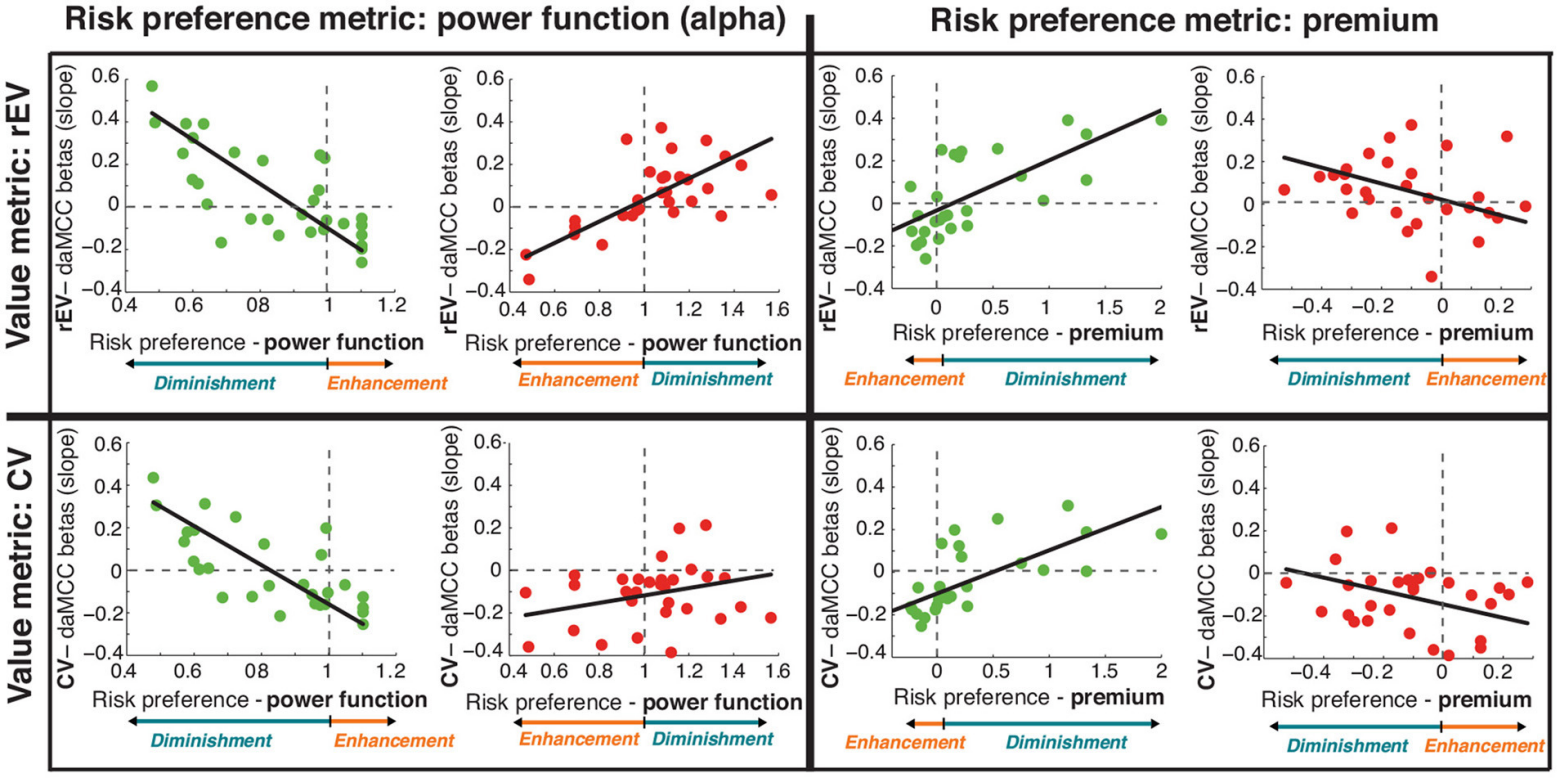
**Relationship between individual preferences and daMCC betas (slopes) across alternative formulations of value and risk preference**. Results from Figures 3C, 4C (in top left cell; gains in green and losses in red) were tested for robustness across a 2 × 2 space. Note that the relationship between risk preference and value subjectification is inverted across gains and losses, and also across the two risk preference measures. For each graph, the relation of the preference metric and value subjectification is shown in color at the bottom. Of note, all eight subplots show the same relationship between daMCC betas and value subjectification—increasing for value diminishment and decreasing for value enhancement.

In our main analyses, we identified a daMCC region as the conjunction of value subjectification across both the gains and losses domains. We performed ROI analyses on this region to visualize the relationship between daMCC beta values and value subjectification. We then repeated this analysis three more times, to produce a 2 × 2 set of analyses, defined by the selected formulation of value on one side and the selected risk preference metric on the other. Within each of these four cells, we examined the relationship independently for the gains and losses domains—resulting in a total of eight scatterplots and correlations (Figure 6).

#### Robustness of value subjectification in the daMCC

In the gains domain, we find significant correlations between daMCC beta values and individual risk preferences for all four pairings of value metrics and risk preference metrics (Figure 6; Table S6). In the losses domain, statistical significance is only present for the initially performed rEV and power function pairing, however, all four of the losses pairings show the same relationship between value subjectification (enhancement or diminishment) and daMCC beta values, with correlation coefficients greater than |.2|. This clear consistency across metrics demonstrates the robustness of the encoding of value subjectification in the daMCC.

### Specificity of the value-to-utility processes: absence during simple mathematical comparisons?

*Post-hoc*, we sought to strengthen the evidence that the daMCC is involved in the value-to-utility transformation by testing the context-specificity of its encoding of the value-to-utility transformation during trials in which the participant should not have been engaging in a conversion from count to worth. This was possible in the trials in which participants chose between two certain options—a comparison that simply requires the identification of the numerically greater value. We note that these trials were randomly intermixed with both the gains and losses trials. We tested whether the information necessary to perform the value-to-utility transformation information was still encoded in the daMCC when no such process was necessary.

Identification of the neural encoding of value-to-utility information during certain vs. certain trials was performed by replicating the rEV regressor for gains and losses within trials with two certain options, for each domain, and covarying the rEV regressor of each domain by the corresponding risk preference of each participant. Whole-brain analyses identified no voxels with significant relations. In addition, ROI analyses were performed on the daMCC ROI, revealing no significant relationship between daMCC beta values and individual risk preference values (gains: *r*_28_ = −0.15, *p* = 0.42; losses: *r*_28_ = 0.16, *p* = 0.41). This analysis indicates that the value-to-utility transformation encoded by the daMCC is context specific, and that this information is not significantly represented outside of the appropriate context. In short, the daMCC encodes the information necessary to compute the value-to-utility transformation that is currently being performed.

### Identifying the neural encoding of parametric losses: no whole-brain significant voxels and no significant encoding in the vmPFC ROI

**Figure 7:**
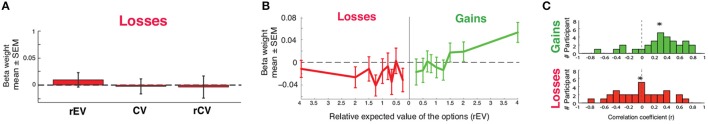
**Neural encoding of value in losses. (A)** ROI analyses measuring the encoding of three different value formulations (relative expected value, rEV; chosen value, CV; relative chosen value, rCV). **(B)** Functional encoding of value in the ventromedial prefrontal cortex (vmPFC), across gains and losses. Beta weights indicate vmPFC activation for each rEV value. **(C)** Distributions of the correlation between each individual's beta weights against the rEV values (^*^mean).

We investigated the neural encoding of linear value signals for losses. For all of three parametric formulations of the value on each trial (rEV, CV, rCV), whole-brain analyses (utilitizing *GLM2a, b, c*) identified no voxels with a significant relationship between reduced activation and reduced value. We tested the encoding of linear loss value signals within the vmPFC through ROI analyses using the same unbiased 10 mm spherical vmPFC ROI (*x* = −2, *y* = 40, *z* = −8) used in gains, extracting the beta values for each value formulation. None of these formulations of loss value were significantly encoded within the vmPFC (*t*_29_ < 0.75, *p* > 0.46) (Figure 7C).

To confirm the absence of a loss value signal in the vmPFC, we extracted and plotted the actual value function encoded within the vmPFC ROI, replicating our previous method in the gains domain. In brief, we extracted the activation for each of the nine rEV levels across losses (utilizing *GLM3*) from the same vmPFC ROI used previously. While gains presented a clear positive monotonic function relating the rEV to brain activation levels within the losses domain the function is flat, showing no relationship between brain activation and value across the rEV levels (Figures 2C, 7B).

As a final step, we investigated the encoding of value signals within each individual, correlating their individual activation for each rEV against the rEV values (separately for gains and losses; Figure 7C). In gains, this correlation analysis revealed a clear positive average correlation in the gains domain (*mean* ± *SD r* = 0.28 ± 0.36, difference from *r* = 0: *t*_29_ = 4.27, *p* < 0.0001). In losses, this correlation analysis revealed a mean-zero distribution with a near uniform distribution, save a large peak at zero (*mean* ± *SD r* = 0.02 ± 0.39, difference from *r* = 0: *t*_29_ = 1.22, *p* = 0.23). While our analyses are able to identify robust encoding of gains value signals within the vmPFC, we find no evidence for the encoding of losses value signals within this region.

## Discussion

The daMCC encodes the context-specific information necessary to perform the value-to-utility transformation, as demonstrated through highly specific modeling and confirmed with in-task replication. Further, we specify that, for a given value, enhanced activation of the daMCC corresponds to value diminishment and deactivation of the daMCC corresponds to value enhancement. These results provide a specific and novel functional role in decision making for the daMCC (also referred to as the aMCC or dmPFC) (Vogt, 2005; Bush, 2009).

Previous studies have implicated the daMCC/dmPFC in decision making, with a range of potential roles from outcome evaluation (Botvinick, 2007), decision conflict (Botvinick et al., 2004; Pochon et al., 2008), reward prediction error (Rushworth and Behrens, 2008), strategic preference (Venkatraman et al., 2009), to degree of uncertainty (Christopoulos et al., 2009). Notably, although Pochon and colleagues beautifully demonstrated that this same region (which they referred to generically as anterior cingulate cortex) is involved in decision making rather than motor planning (Pochon et al., 2008), their task and analyses could not differentiate between a number of co-occurring cognitive processes, ranging from attention, memory, theory of mind, and even face processing. In fact, their full results suggest that their decision conflict analysis may have reflected all of these cognitive processes, as they identified parametric encoding of decision conflict in numerous brain regions (including the dorsolateral prefrontal cortex, parahippocampal gyrus, fusiform gyrus, and striate visual area; Pochon et al., 2008). Further, choice difficulty is not a cognitive process or computation, but rather is a comparison of task states that reflects the need for greater computations to compare across options to reach a decision (i.e., a comparative need for greater processing), but does not identify what those processes are. In contrast, the high precision of our between-subject covariate analyses (a 270-point fit [9 levels of rEV covaried by the preference values of 30 participants], with 15 trials per point) safeguards our results from being the result of co-occurring cognitive processes—the specificity of the computational model allows specificity of the identified cognitive processes. We note explicitly that our results cannot be due to choice difficulty, as the value-to-utility functions are orthogonal to trial-by-trial choice difficulty (as both a within- and between-subject fit of trial-by-trial value covaried by individual preference). Our precise analyses suggest a specific computation that is occurring within the daMCC—that the daMCC encodes the information necessary to perform the value-to-utility transformation. One possible reconciliation is that choice difficulty reflects greater required precision of the value-to-utility transformation, in which case our results can readily explain those found by Pochon et al. (2008).

We note that our results should not be interpreted in an exclusionary manner. We do not provide evidence that the daMCC does not encode other information, or is uninvolved in other functions. In fact, in serving the computation of the value-to-utility transformation this region is likely to encode a myriad of factors that could be integrated to determine the context-specific transformation. This could explain the wealth of studies that have found this region modulated by risk preference, degree of uncertainty, and even effort-related costs (Klein-Flügge et al., 2016). We hypothesize that this is likely to extend beyond economic factors to social and even moral domains. The specific components present are likely to be highly context specific, and the critical feature is not which ones are currently present in any given study (which will depend on the task), but that they come together in an orderly fashion to be able to compute the value-to-utility transformation. Quite simply, the whole is greater than the sum of the parts.

Even with the high specificity of our analyses, they cannot provide causal evidence of the role of the daMCC in the value-to-utility transformation. However, a recent study has indicated that activation patterns within a proximal dmPFC region can predict risky decision making prior to the presentation of the available options (Huang et al., 2014). The predictive power of this information is suggestive of causal evidence—that is, varied activation patterns within the daMCC can modulate how not-yet-presented stimuli will be judged. Our study suggests an intriguing mechanism for this predicted choice behavior: that fluctuation in the daMCC prior to option presentation may modulate the value-to-utility transformation of the incoming options, altering their computed utilities and therefore biasing choice behavior.

The functional connectivity analyses indicate a functional network of regions with which the daMCC communicates during risky decision making—the IFG and NAcc. Although these analyses cannot inform on the directionality of signals, given the known role of the NAcc in valuation (Knutson et al., 2001, 2005; Abler et al., 2006; Peters and Büchel, 2010), and IFG's role in executive processing and working memory (Duncan and Owen, 2000; Mullette-Gillman and Huettel, 2009), we hypothesize that the value-to-utility transformation within the daMCC is “set” contextually by inputs from the IFG, and outputs to modulate value signals within the NAcc.

We see an excellent substrate for the value-to-utility transformation in the interactions of the daMCC and NAcc, combining across the value-to-utility transformation covariate analyses and the functional connectivity analyses. Our covariate analyses demonstrate that the daMCC activation has a zero-centered negative relationship with the degree of the value-to-utility transformation—daMCC activation results in reduced subjective valuation, daMCC deactivation results in enhanced subjective valuation, and baseline daMCC activation (non-modulated) results in non-modulated subjective valuation (i.e., subjective value = objective value). Numerous studies have shown that the activity of the NAcc is positively correlated with reward value (Knutson et al., 2001, 2005; Peters and Büchel, 2010). The negative functional connectivity identified between the daMCC and NAcc, and the presence of value signals within the NAcc, combine to suggest that inhibitory or excitatory signals from the daMCC to NAcc could drive altered subjective valuation. Simply, our results suggest that subjective valuation could be the result of daMCC activation inversely modulating valuative processes in the NAcc. From the NAcc, these modulated valuation signals (subjective value or utility) may be transmitted to other regions that have been found to encode value signals, such as the vmPFC and striatum (Bartra et al., 2013; Clithero and Rangel, 2014).

Our experiment suggests multiple hypotheses about the nature of the populations of neurons encoding the value-to-utility transformation. First, it appears that this system has a fast temporal profile for setting the context-specific value-to-utility transformation, as we show independence for gains and losses risk preferences even though trials were randomly intermixed, occurred every few seconds, and the value-to-utility information for each domain is encoded in largely overlapping brain regions. Secondly, it appears that this region has a low inertia, as we find that neither gains or losses preference values are encoded during trials in which risk was absent (when the participants selected between two certain options). Finally, our third hypothesis is that simultaneous value-to-utility transformations engaging overlapping neural populations will lead to a neural bottleneck, resulting in computational interference and reduced decision-making quality. Within this study we see that the value-to-utility transformations for gains and losses are encoded in largely overlapping neural region, even though their transformation values (α risk preference values) are uncorrelated. Should this overlap continue to the neuronal level, then it is possible that this will lead to interference in neural processing, resulting in a blending of the informational signals being processed—in this case the value-to-utility transformation parameter. For example, consider a participant that strongly diminishes the utility of potential gains (highly risk averse) and weakly enhances the utility of a potential loss (risk seeking). If this hypothesis is true, then when this individual is presented with a gamble that contains both a possible gain and a possible loss, they may show preferences that are a blending (average) of the two independent preferences—weak diminishment of utility (weak risk aversion) for both gains and losses. As a further example, let us consider the ubiquitous use of sexual images to sell consumer products. While it is certainly the case that the attractiveness of a model on a car provides no information about the quality of the car under consideration, the presence of such an evolutionarily predisposed signal may lead to artificial modulation of the value-to-utility transformation of the consumer good (car, clothing, etc.). We believe that it will be of great interest to examine how coincident value-to-utility transformations are processed within this region, and specifically whether simultaneous value-to-utility transformations are independent (as would be the case for a computer) or lead to computational interference.

### Ancillary finding: absence of value signals for losses

No brain regions demonstrated whole-brain significance for any of the three formulations of linear value signals for losses during the replication. Further analyses demonstrated that within the vmPFC ROI (derived from meta-analyses on value signaling in the gains domain) there was no modulation of activation levels across the nine levels of losses valuation examined in the rEV formulation. While many neuroimaging experiments have examined value coding in gains, with consistent evidence that gains value signals are encoded within the vmPFC (see recent meta-analyses, Bartra et al., 2013; Clithero and Rangel, 2014), only a few have explored this issue in losses and recent meta-analyses have not found evidence that the vmPFC also encodes losses. Within the few experiments that have directly examined value coding for losses, the methods of these experiments result in some ambiguities on how to best interpret their findings. For example, Litt et al. (2011) asked participants “How much would you like to eat this item…” and encoded responses of “Not at all” as the highest level of loss. It may be that such responses would be more appropriately considered as low gains, and, if their results are re-interpreted along these lines then their results show linear encoding from low gains to high gains within the vmPFC (concurring with numerous studies), and unfortunately their results would no longer address how losses are encoded.

Similar issues arise in a study by Tom et al. (2007), which identified neural encoding of the possible losses value within the vmPFC through whole-brain analyses. We see two possible explanations for the contrast with our lack of such encoding. First, their task featured both possible gains and possible losses on each trial (choice of whether to engage in a 50:50 gamble, with possible gain and loss), and it is possible that the vmPFC does not encode losses in isolation but may contain information about possible losses when they are being contrasted to the possible gains (or some other manner of interaction). Secondly, it is possible that participants framed all possible outcomes as gains. This possibility comes from the experimental design, as participants were given $20 prior to the task and then all trials featured potential losses up to that $20 value. The goal was that participants would make decisions relative to the $20, but it is possible that they instead made decisions framed by the final possible outcome instead (i.e., a 50:50 gamble with a possible gain of $10 and a possible loss of $5, incorporates the $20 to become a 50:50 possible gain of $30 or a possible gain of $15). We believe that our task design avoids this issue, as we feature a possible zero outcome in all gambles—providing a simple common frame across both gains and losses trials. There is clear evidence of large behavioral differences between gains and losses decision making (see Kurnianingsih and Mullette-Gillman, 2015), which could be due to divergent neural processing for gains and losses. Here we find evidence of significant differences in neural processing of gains (numerous regions identified in whole-brain analyses and clear value function encoding in the vmPFC) and losses (whole-brain null effect and the subsequent flat value function in the vmPFC ROI), suggesting the need for further neuroimaging studies on the neural basis of loss value coding.

## Conclusions

We identified the neural instantiation of the value-to-utility transformation within the daMCC. Further, we describe a simple network of regions through which contextual information (contingency and long-term history) could be integrated to determine the degree and direction of the value-to-utility transformation, and through which modulatory signals could output to alter valuation signals.

## Author contributions

OM conceived the experiment; OM and YK designed the experiment; YK collected the data under the supervision of OM; YK, and OM analyzed the data and wrote the manuscript.

### Conflict of interest statement

The authors declare that the research was conducted in the absence of any commercial or financial relationships that could be construed as a potential conflict of interest.
