# Supplementary material for: Neural Mechanisms of the Transformation from Objective Value to Subjective Utility: Converting from Count to Worth
Source: Front Neurosci. 2016 Nov 9;10:507. doi: 10.3389/fnins.2016.00507 (PMC5101215; doi:10.3389/fnins.2016.00507)
Supplement: Supplementary file 1 [file Table1.docx]

Supplementary Material

**Converting from count to worth: Neural mechanisms of the transformation from objective value to subjective utility**

Yoanna A. Kurnianingsih^1^ and O’Dhaniel A. Mullette-Gillman^1,2,3*^

^1^Department of Psychology, National University of Singapore, Singapore 117570, Singapore; ^2^Neuroscience and Behavioral Disorders Program, Duke-NUS Medical School, Singapore 169857, Singapore; ^3^SINAPSE Institute for Neurotechnologies, National University of Singapore, Singapore 117456, Singapore

^*^**Correspondence**: odubik@gmail.com

Department of Psychology, FASS, NUS

Block AS4, #02-07, 9 Arts Link, Singapore 117570

# Supplementary Tables

**Table S1.** **Median correlations between parametric regressors across runs**

| Gains  Median (SD) | | RC Trials | | | CC Trials | | Losses  Median (SD) | | RC Trials | | | CC Trials | |
| --- | --- | --- | --- | --- | --- | --- | --- | --- | --- | --- | --- | --- | --- |
|  |  | rEV | rCV | CV | rEV | rCV |  |  | rEV | rCV | CV | rEV | rCV |
| RC Trials | rCV | .357  (.234) |  |  |  |  | RC Trials | rCV | -.348  (.288) |  |  |  |  |
|  | CV | .831  (.138) | .537  (.085) |  |  |  |  | CV | -.413  (.121) | -.398  (.198) |  |  |  |
|  | pWIN | .003  (.105) | .083  (.120) | .033  (.152) |  |  |  | pWIN | -.024  (.099) | -.169  (.146) | .082  (.109) |  |  |
| CC Trials | rCV |  |  |  | 1.000  (.035) |  | CC Trials | rCV |  |  |  | .956  (.256) |  |
|  | CV |  |  |  | .831  (.138) | .513  (.117) |  | CV |  |  |  | .249  (.201) | -.259  (.219) |

Abbreviations: RC, risky vs. certain; CC, certain vs. certain; rEV, relative expected value; rCV, relative chosen value; CV, chosen value; pWIN, probability of winning.

**Table S2.** **GLM1: Brain areas exhibiting significant differences in activation across trial types within each domain**

|  |  | # voxels | Region | Hemisphere | Peak Coordinates | | | *z-stat* |
| --- | --- | --- | --- | --- | --- | --- | --- | --- |
|  |  |  |  |  | *x* | *y* | *z* |  |
| **Gains** | **RC > CC** | 13817 | Paracingulate Gyrus  (*dmPFC) | L | -2 | 34 | 32 | 5.75 |
|  |  |  |  | mid | 0 | 24 | 46 | 5.65 |
|  |  |  | Inferior Frontal Gyrus | L | -50 | 18 | 28 | 5.44 |
|  |  | 26578 | Lateral Occipital Cortex | L | -38 | -88 | 8 | 6.99 |
|  |  |  |  | L | -34 | -86 | 8 | 6.82 |
|  |  |  |  | L | -34 | -82 | -14 | 6.74 |
|  |  | 2461 | Posterior Cingulate Gyrus | L | -2 | -24 | 28 | 5.21 |
|  |  |  |  | mid | 6 | -30 | 26 | 4.58 |
|  |  |  | Caudate | R | 14 | 20 | -4 | 4.16 |
|  | **CC > RC** | 1044 | Frontal Medial Cortex | L | -2 | 48 | -20 | 4.13 |
|  |  |  | (*vmPFC) | L | -6 | 40 | -22 | 3.73 |
|  |  |  | Frontal Pole | R | 4 | 60 | 6 | 3.53 |
|  |  | 2422 | Supramarginal Gyrus | R | 62 | -36 | 28 | 5.85 |
|  |  |  |  | R | 62 | -44 | 34 | 5.35 |
|  |  |  |  | R | 62 | -26 | 22 | 5.12 |
|  |  | 1865 | Supramarginal Gyrus | L | -64 | -34 | 26 | 5.02 |
|  |  |  |  | L | -66 | -26 | 26 | 4.81 |
|  |  |  |  | L | -64 | -42 | 42 | 4.42 |
|  |  | 809 | Postcentral Gyrus | R | 12 | -38 | 50 | 4.06 |
|  |  |  | Anterior Cingulate Gyrus | mid | 0 | -14 | 40 | 3.99 |
|  |  |  | Posterior Cingulate Gyrus | R | 10 | -28 | 40 | 3.67 |
| **Losses** | **RC > CC** | 23397 | Lateral Occipital Cortex | L | -34 | -90 | 12 | 6.39 |
|  |  |  |  | R | 28 | -64 | 52 | 5.84 |
|  |  |  | Middle Temporal Gyrus | R | 40 | -58 | 46 | 6.09 |
|  |  | 17405 | Inferior Frontal Gyrus | L | -4 | 26 | 38 | 6.53 |
|  |  |  | Posterior Cingulate Gyrus | L | -2 | 20 | 44 | 6.12 |
|  |  |  | Frontal Pole | L | -40 | 48 | 0 | 5.66 |
|  |  | 4092 | Lateral Ventricle | R | 6 | 0 | 22 | 4.56 |
|  |  |  | Thalamus | L | -20 | -30 | 2 | 4.41 |
|  |  |  |  | R | 16 | -18 | 0 | 4.15 |
|  | **CC > RC** | 1338 | Supramarginal Gyrus | R | 66 | -36 | 32 | 4.99 |
|  |  |  |  | R | 64 | -42 | 36 | 4.69 |
|  |  |  |  | R | 66 | -44 | 28 | 4.65 |
|  |  | 1191 | Frontal Medial Cortex  (*vmPFC) | L | -10 | 50 | -10 | 4.46 |
|  |  |  | Cingulate Gyrus | L | 4 | 36 | -8 | 4.28 |
|  |  |  | Subcallosal Cortex | L | -4 | 24 | -16 | 4.1 |
|  |  | 969 | Supramarginal Gyrus | L | -66 | -28 | 26 | 4.57 |
|  |  |  |  | L | -60 | -28 | 24 | 4.17 |
|  |  |  |  | L | -62 | -40 | 36 | 4.07 |

Abbreviations: RC, risky vs. certain; CC, certain vs. certain; vmPFC, ventromedial prefrontal cortex; dmPFC, dorsomedial prefrontal cortex.

Regions are labeled based upon their Harvard-Oxford Atlas designations, with parenthetical inclusion of labels from text.

The coordinates for the three peak activations are provided for each cluster, in MNI space (in mm).

**Table S3.** **GLM1: Brain areas exhibiting significant difference in activation across the gains and losses domains, within each trial type**

|  |  | #  voxels | Region | Hemisphere | Peak Coordinates | | | *z-stat* |
| --- | --- | --- | --- | --- | --- | --- | --- | --- |
|  |  |  |  |  | *x* | *y* | *z* |  |
| **CC** | **Losses > Gains** | 443 | Caudate | R | 16 | 26 | -8 | 4.04 |
|  |  |  |  | R | 22 | 28 | 4 | 3.5 |
|  |  |  |  | R | 4 | 16 | -4 | 3.47 |
|  |  | 449 | Inferior Frontal Gyrus | R | 48 | 10 | 22 | 3.41 |
|  |  |  |  | R | 30 | 14 | 20 | 3.32 |
|  |  |  |  | R | 42 | 0 | 20 | 3.28 |
|  |  | 4867 | Lateral Occipital Cortex | L | -42 | -90 | -8 | 6.27 |
|  |  |  | Occipital Pole | L | -34 | -94 | 2 | 5.42 |
|  |  |  |  | L | -20 | -94 | -14 | 5.4 |
|  |  | 5095 | Occipital Pole | R | 34 | -92 | -4 | 5.55 |
|  |  |  | Lateral Occipital Cortex | R | 32 | -88 | -14 | 5.21 |
|  |  |  | Occipital Fusiform Gyrus | R | 38 | -72 | -16 | 5.06 |
| **RC** | **Gains > Losses** | 435 | Frontal Medial Cortex | mid | 0 | 42 | -14 | 3.58 |
|  |  |  | (*vmPFC) | R | 8 | 40 | -22 | 3.37 |
|  |  |  | Frontal Pole | R | 12 | 40 | -20 | 3.34 |
| **RC** | **Losses >**  **Gains** | 11059 | Inferior Frontal Gyrus | R | 44 | 12 | 26 | 5.6 |
|  |  |  |  | L | -40 | 10 | 22 | 4.79 |
|  |  |  | Paracingulate Gyrus (*dmPFC) | mid | 0 | 16 | 46 | 4.6 |
|  |  | 890 | Thalamus | R | 8 | -14 | 8 | 3.83 |
|  |  |  |  | R | 18 | -10 | 18 | 3.5 |
|  |  |  |  | L | -20 | -30 | 8 | 3.25 |
|  |  | 17479 | Occipital Pole | L | -26 | -98 | -8 | 5.55 |
|  |  |  | Occipital Fusiform Gyrus | R | 30 | -86 | -10 | 5.51 |
|  |  |  | Hippocampus | L | -34 | -34 | -90 | 5 |

Abbreviations: RC, risky vs. certain; CC, certain vs. certain; vmPFC, ventromedial prefrontal cortex; dmPFC, dorsomedial prefrontal cortex.

Regions are labeled based upon their Harvard-Oxford Atlas designations, with parenthetical inclusion of labels from text.

The coordinates for the three peak activations are provided for each cluster, in MNI space (in mm).

**Table S4. GLM2: Brain areas exhibiting significant encoding of parametric value signals in the gains domain**

|  | # voxels | Region | Hemisphere | Peak Coordinates | | | *z-stat* |
| --- | --- | --- | --- | --- | --- | --- | --- |
|  |  |  |  | *x* | *y* | *z* |  |
| **rEV** | 2474 | Frontal Medial Cortex  (*vmPFC) | R | 4 | 50 | -8 | 3.97 |
|  |  |  | R | 6 | 40 | -14 | 3.91 |
|  |  | Paracingulate Gyrus | R | 4 | 44 | -8 | 3.91 |
|  | 533 | Posterior Cingulate Gyrus  (*PCC) | R | 6 | -30 | 30 | 3.65 |
|  |  |  | R | 4 | -34 | 36 | 3.63 |
|  |  |  | L | -2 | -36 | 44 | 3.62 |
|  | 473 | Middle Temporal Gyrus | R | 58 | -56 | -6 | 3.68 |
|  |  |  | R | 62 | -54 | -6 | 3.66 |
|  |  | Inferior Temporal Gyrus | R | 52 | -50 | -14 | 3.46 |
|  | 528 | Lateral Occipital Cortex | R | 24 | -82 | 50 | 3.49 |
|  |  |  | R | 26 | -60 | 42 | 3.29 |
|  |  |  | R | 32 | -66 | 46 | 3.28 |
|  | 2381 | Occipital Pole | L | -20 | -102 | 0 | 4.29 |
|  |  | Lateral Occipital Cortex | L | -40 | -90 | 16 | 3.84 |
|  |  |  | L | -34 | -86 | 10 | 3.83 |
| **CV** | 1107 | Frontal Medial Cortex (*vmPFC) | R | 6 | 40 | -14 | 4 |
|  |  |  | R | 2 | 44 | -8 | 3.83 |
|  |  |  | L | -10 | 38 | -12 | 3.47 |
|  | 1128 | Occipital Pole | L | -20 | -102 | 0 | 3.73 |
|  |  |  | L | -26 | -98 | 6 | 3.4 |
|  |  | Lateral Occipital Cortex | L | -22 | -76 | 52 | 3.39 |

Abbreviations: rEV, relative expected value; CV, chosen value; vmPFC, ventromedial prefrontal cortex; PCC, posterior cingulate cortex.

Regions are labeled based upon their Harvard-Oxford Atlas designations, with parenthetical inclusion of labels from text.

The coordinates for the three peak activations are provided for each cluster, in MNI space (in mm).

**Table S5. Brain areas exhibiting significant functional connectivity with the daMCC seed region during the decision period of risky vs. certain trials within each domain**

|  |  | # voxels | Region | Hemisphere | Peak Coordinates | | | *z-stat* |
| --- | --- | --- | --- | --- | --- | --- | --- | --- |
|  |  |  |  |  | *x* | *y* | *z* |  |
| **Gains** | **Positive** | 286 | Inferior Frontal Gyrus | L | -44 | 28 | 18 | 3.56 |
|  |  |  |  | L | -40 | 30 | 10 | 3.45 |
|  |  |  |  | L | -38 | 20 | 16 | 3.23 |
|  | **Negative** | 390 | Angular Gyrus | R | 38 | -56 | 44 | 4.03 |
|  |  |  | Lateral Occipital Cortex | R | 40 | -62 | 52 | 3.79 |
|  |  |  |  | R | 36 | -60 | 56 | 3.2 |
|  |  | 2568 | Occipital Pole | R | 6 | -92 | 6 | 4.43 |
|  |  |  |  | L | -6 | -100 | 0 | 4.29 |
|  |  |  |  | L | -8 | -98 | 6 | 4.2 |
| **Losses** | **Positive** | 359 | Inferior Frontal Gyrus | L | -38 | 24 | 20 | 3.54 |
|  |  |  |  | L | -52 | 18 | 30 | 3.52 |
|  |  |  |  | L | -46 | 28 | 18 | 3.1 |
|  | **Negative** | 347 | Frontal Orbital Cortex | L | -20 | 16 | -14 | 3.36 |
|  |  |  |  | L | -14 | 16 | -12 | 3.22 |
|  |  |  | Putamen | L | -26 | -2 | 2 | 3.34 |
|  |  | 513 | Lateral Occipital Cortex | R | 34 | -62 | 48 | 3.62 |
|  |  |  |  | R | 42 | -60 | 46 | 3.45 |
|  |  |  |  | R | 38 | -62 | 50 | 3.39 |
|  |  | 1847 | Occipital Pole | L | 8 | -100 | 0 | 4.09 |
|  |  |  |  | L | -6 | -94 | 14 | 3.98 |
|  |  |  | Lingual Gyrus | L | -2 | -74 | 2 | 4.07 |

Regions are labeled based upon their Harvard-Oxford Atlas designations.

The coordinates for the three peak activations are provided for each cluster, in MNI space (in mm).

**Table S6. Testing the robustness of value-to-utility transformation encoding in the daMCC: Correlations between dmPFC ROI beta values and individual preferences, across two formulations of value (rEV and CV) and two measures of risk preference (power function and premium)**

|  |  | Risk Preference Metric | |
| --- | --- | --- | --- |
|  |  | Power Function | Premium |
| [Value]  regressor  formulation | rEV | Gains: *r* = -.73, *p* < .0001  Losses: *r* = .64, *p* < .0001 | Gains: *r* = .68, *p* < .0001  Losses: *r* = -.22, *p* = .24 |
|  | CV | Gains: *r* = -.75, *p* < .0001  Losses: *r* = .22, *p* = .24 | Gains: *r* = .65, *p* < .0001  Losses: *r* = -.23, *p* = .21 |

Abbreviations: rEV, relative expected value; CV, chosen value.

Expanding from rEV [Value] regressor covaried by power function risk preference metric (top left cell of correlations) to 2**×**2 of [Value] by [Risk Preference].
